# Supplementary material for: ProteinShader: illustrative rendering of macromolecules
Source: BMC Struct Biol. 2009 Mar 30;9:19. doi: 10.1186/1472-6807-9-19 (PMC2672931; doi:10.1186/1472-6807-9-19)
Supplement: Additional file 1 — ProteinShader program without source code. This compressed file contains the complete ProteinShader program including associated libraries, but no source code. A README.txt file gives an overview of the ProteinShader distribution, and the index.html file in the help subdirectory has directions on getting started with the program as well as a set of tutorials. [file 1472-6807-9-19-S1.zip › ProteinShader-beta-0_9_4-binary/help/api/org/proteinshader/structure/visitor/exceptions/class-use/VisitorException.html]

Uses of Class org.proteinshader.structure.visitor.exceptions.VisitorException (ProteinShader API)


|  |  |  |
| --- | --- | --- |
| **Overview** \| **Package** \| **Class** \| **Use** \| **Tree** \| **Deprecated** \| **Index** \| **Help** | |  |
| PREV   NEXT | **FRAMES**    **NO FRAMES**     **All Classes** |  |


---


## **Uses of Class org.proteinshader.structure.visitor.exceptions.VisitorException**

| Packages that use VisitorException | |
| --- | --- |
| **org.proteinshader.gui** | Holds all of the Swing GUI components and their associated listeners, including class Renderer, which is registered as a listener for the GLCanvas object that is used a drawing surface. |
| **org.proteinshader.structure** | Holds the classes that store information from a Protein Data Bank file: Structure, Model, Chain, AminoAcid, Heterogen, Water, Atom, Bond, Helix, BetaStrand, Loop, *etc*. |
| **org.proteinshader.structure.io.demos** | Holds demo classes that were used for early testing of the io classes. |
| **org.proteinshader.structure.visitor** | Holds Visitor classes that know how to traverse the hierarchy of objects held by class Structure. |
| **org.proteinshader.structure.visitor.demos** | Holds demo classes that were used for early testing of the Visitor classes. |
| **org.proteinshader.structure.visitor.exceptions** | Holds exception classes needed by the Visitor classes. |

Uses of VisitorException in org.proteinshader.gui

| Methods in org.proteinshader.gui that throw VisitorException | |
| --- | --- |
| `void` | `Renderer.setVisibleDrawables(Model model, StyleEnum style)`             An array of opaque Segments, and array of opaque Atoms and Bonds, and an array of translucent Drawable objects will be obtained from the Model. |

Uses of VisitorException in org.proteinshader.structure

| Methods in org.proteinshader.structure that throw VisitorException | |
| --- | --- |
| `void` | `AminoAcid.accept(Visitor visitor)`             Accepts a Visitor and does a callback. |
| `void` | `Water.accept(Visitor visitor)`             Accepts a Visitor and does a callback. |
| `abstract  void` | `Drawable.accept(Visitor visitor)`             Accepts a Visitor and does a callback. |
| `void` | `Segment.accept(Visitor visitor)`             Accepts a Visitor and does a callback. |
| `void` | `Structure.accept(Visitor visitor)`             Accepts a Visitor and does a callback. |
| `abstract  void` | `Residue.accept(Visitor visitor)`             Accepts a Visitor and does a callback. |
| `void` | `Chain.accept(Visitor visitor)`             Accepts a Visitor and does a callback. |
| `void` | `Model.accept(Visitor visitor)`             Accepts a Visitor and does a callback. |
| `void` | `Helix.accept(Visitor visitor)`             Accepts a Visitor and does a callback. |
| `void` | `Atom.accept(Visitor visitor)`             Accepts a Visitor and does a callback. |
| `abstract  void` | `Region.accept(Visitor visitor)`             Accepts a Visitor and does a callback. |
| `void` | `BetaStrand.accept(Visitor visitor)`             Accepts a Visitor and does a callback. |
| `void` | `Loop.accept(Visitor visitor)`             Accepts a Visitor and does a callback. |
| `void` | `Bond.accept(Visitor visitor)`             Accepts a Visitor and does a callback. |
| `void` | `Heterogen.accept(Visitor visitor)`             Accepts a Visitor and does a callback. |
| `void` | `Structure.calculateBounds()`             Sets the min and max values for x, y, and z on each Model held by the Structure. |
| `void` | `Structure.calculateFrenetFrames()`             Before Regions objects (Helix, BetaStrand, and Loop) are added to the Structure, a rotation (the equivalent to a discrete Frenet Frame) should be calculated for each AminoAcid. |
| `void` | `Structure.clearAllBonds()`             Clears all Bonds (AminoAcids, Heterogens, and Waters) from the Structure. |
| `void` | `Structure.clearBonds(ResidueMode residueMode)`             Clears Bonds from the Structure. |
| `void` | `Structure.generateLoopRegions()`             Generates Loop objects from any AminoAcids that do not already belong to a Helix or BetaStrand object. |
| `void` | `Structure.generateStandardBonds()`             Generates standard Bonds (AminoAcids and Waters). |
| `List<String>` | `Structure.getAminoAcidLabels()`             Returns a list of all AminoAcid residueIDs. |
| `void` | `Model.updateListsOfVisibles(boolean includeAtoms, boolean includeBonds, boolean includeAminoAcids, boolean includeHeterogens, boolean includeWaters, boolean includeSegments)`             Finds visible Drawable objects and transfers them to a list of opaque Segments, opaque Atoms and Bonds, or a list of translucent Drawables. |

Uses of VisitorException in org.proteinshader.structure.io.demos

| Methods in org.proteinshader.structure.io.demos that throw VisitorException | |
| --- | --- |
| `void` | `PDBStructureReaderDemo.writeStructure(Structure structure, String filename)`             Writes a Structure to a file by using a SFWriterVisitor. |
| `void` | `SegmentDemo.writeStructure(Structure structure, String filename)`             Writes a Structure to a file by using a SFWriterVisitor. |

Uses of VisitorException in org.proteinshader.structure.visitor

| Methods in org.proteinshader.structure.visitor that throw VisitorException | |
| --- | --- |
| `void` | `Visitable.accept(Visitor visitor)`             Accepts a Vistor so that a call back can be done. |
| `void` | `SFWriterVisitor.closeOutputFile()`             This method flushes the output buffer to make sure that all data is written before closing the output file. |
| `void` | `SFWriterVisitor.flush()`             This method forces the writer to immediately write all data in its buffer. |
| `void` | `SFWriterVisitor.openOutputFile(String outputFile)`             Opens a buffered writer using the filename given as an argument. |
| `void` | `Visitor.visit(AminoAcid aminoAcid)`             Traverses all Atoms owned by the AminoAcid if AAPortionMode is set to ENTIRE\_AA. |
| `void` | `SFWriterVisitor.visit(AminoAcid aminoAcid)`             Writes all information held by an AminoAcid. |
| `void` | `FrenetFrameGeneratorVisitor.visit(AminoAcid aminoAcid)`             The AminoAcid being visited is considered as CA(i+1), while the last two AminoAcids are considered to be CA(i) and CA(i-1). |
| `void` | `LoopGeneratorVisitor.visit(AminoAcid aminoAcid)`             Checks whether a start AminoAcid and an end AminoAcid for a Loop have been found, and creates a new Loop as needed. |
| `void` | `AminoAcidLabelVisitor.visit(AminoAcid aminoAcid)`             Adds a residueID to the list of labels if it has not been seen before. |
| `void` | `BondGeneratorVisitor.visit(AminoAcid aminoAcid)`             Uses Atom distances to generate Bonds within the AminoAcid, and also uses the memory of the last carbonyl carbon seen to check for a possible peptide Bond. |
| `void` | `ModifierVisitor.visit(Atom atom)`             If an AtomModifier has been added, it will be used to modify the Atom. |
| `void` | `Visitor.visit(Atom atom)`             Traverses all Bonds owned by the Atom. |
| `void` | `SFWriterVisitor.visit(Atom atom)`             Writes all information held by an Atom. |
| `void` | `BoundsVisitor.visit(Atom atom)`             Checks xyz-coordinates to look for any new min or new max. |
| `void` | `VisibilityVisitor.visit(Atom atom)`             Tests the visiblity of the Atom in order to determine if it should be transferred to the opaque Atoms and Bonds list or the translucent Drawables list. |
| `void` | `BondDestroyerVisitor.visit(Atom atom)`             Removes all Bonds on the Atom. |
| `void` | `BondGeneratorVisitor.visit(Atom atom)`             Checks if the Atom is a possible Bond destination Atom for any previously seen Atoms of the same Residue, and then adds the Atom to the list of Atoms to remember. |
| `void` | `Visitor.visit(BetaStrand betaStrand)`             If debug is true, the betaStrandID, startResidueID, and endResidueID will be printed to standard out. |
| `void` | `SFWriterVisitor.visit(BetaStrand betaStrand)`             Writes all information held by a BetaStrand. |
| `void` | `ModifierVisitor.visit(Bond bond)`             If a BondModifier has been added, it will be used to modify the Bond. |
| `void` | `Visitor.visit(Bond bond)`             If debug is true, the atomID of both the source Atom and the destination Atom will be printed to standard out. |
| `void` | `SFWriterVisitor.visit(Bond bond)`             Writes all information held by a Bond. |
| `void` | `VisibilityVisitor.visit(Bond bond)`             Tests the visiblity of the Bond in order to determine if it should be transferred to the opaque Atoms and Bonds or translucent Drawables list. |
| `void` | `BondGeneratorVisitor.visit(Bond bond)`             NO OPERATION: This Visitor method is not needed by the bond generator. |
| `void` | `Visitor.visit(Chain chain)`             Uses the current ResidueMode to determine if it should traverse the AminoAcids, Heterogens, and/or Waters owned by the Chain. |
| `void` | `SFWriterVisitor.visit(Chain chain)`             Writes all information held by a Chain. |
| `void` | `FrenetFrameGeneratorVisitor.visit(Chain chain)`             All AminoAcids of the Chain will be visited. |
| `void` | `LoopGeneratorVisitor.visit(Chain chain)`             Generates Loop objects from AminoAcids that do not already belong to any Region. |
| `void` | `BondGeneratorVisitor.visit(Chain chain)`             Generates all standard Bonds (AminoAcids and Waters) for the Chain. |
| `void` | `Visitor.visit(Helix helix)`             If debug is true, the helixID, startResidueID, and endResidueID will be printed to standard out. |
| `void` | `SFWriterVisitor.visit(Helix helix)`             Writes all information held by a Helix. |
| `void` | `Visitor.visit(Heterogen heterogen)`             Traverses all Atoms owned by the Heterogen. |
| `void` | `SFWriterVisitor.visit(Heterogen heterogen)`             Writes all information held by a Heterogen. |
| `void` | `BondGeneratorVisitor.visit(Heterogen heterogen)`             NO OPERATION: This method is not needed because non-water heterogen bonds are given explicitly in the PDB structure entry. |
| `void` | `Visitor.visit(Loop loop)`             If debug is true, the loopID will be printed to standard out. |
| `void` | `SFWriterVisitor.visit(Loop loop)`             Writes all information held by a Loop. |
| `void` | `Visitor.visit(Model model)`             Traverses all Chains owned by the Model. |
| `void` | `SFWriterVisitor.visit(Model model)`             Writes all information held by a Model. |
| `void` | `BoundsVisitor.visit(Model model)`             Finds the min and max values for x, y, and z of the Model and then calls setter methods on the Model so that these values are remembered. |
| `void` | `LoopGeneratorVisitor.visit(Model model)`             Generates all Loop objects needed by the Model. |
| `void` | `BondGeneratorVisitor.visit(Model model)`             Generates all standard Bonds (AminoAcids and Waters) for the Model. |
| `void` | `ModifierVisitor.visit(Segment segment)`             If a SegmentModifier has been added, it will be used to modify the Segment. |
| `void` | `Visitor.visit(Segment segment)`             If debug is true, the segmentID will be printed to standard out. |
| `void` | `SFWriterVisitor.visit(Segment segment)`             Writes information on a Segment. |
| `void` | `VisibilityVisitor.visit(Segment segment)`             Tests the visiblity of the Segment in order to determine if it should be transferred to the opaque Segments or translucent Drawables list. |
| `void` | `Visitor.visit(Structure structure)`             Traverses all Models owned by the Structure. |
| `void` | `SFWriterVisitor.visit(Structure structure)`             Writes a Structure's data to a simple text file. |
| `void` | `BondGeneratorVisitor.visit(Structure structure)`             Generates all standard Bonds (AminoAcids and Waters) for the Structure. |
| `void` | `Visitor.visit(Water water)`             Traverses all Atoms owned by the Water. |
| `void` | `SFWriterVisitor.visit(Water water)`             Writes all information held by a Water. |
| `void` | `BondGeneratorVisitor.visit(Water water)`             If the Water includes hydrogen atoms, then Bonds will be created between the oxygen and each hydrogen (after checking that the Bond lengths are reasonable). |

Constructors in org.proteinshader.structure.visitor that throw VisitorException

`SFWriterVisitor(String outputFile)`
  
          Constructs a SFWriterVisitor that will write to the output file
given as an argument.

Uses of VisitorException in org.proteinshader.structure.visitor.demos

| Methods in org.proteinshader.structure.visitor.demos that throw VisitorException | |
| --- | --- |
| `void` | `ModifierVisitorDemo.modify(Structure structure)`             The Helices of the Structure are modified by changing their shape to CYLINDER and setting all of their Atoms and Bonds to INVISIBLE. |
| `void` | `VisitorDemo.testVisitor(Structure structure)`             Creates a Visitor and tests it by activating debug mode so that it will print the ID of each object in the Structure. |
| `void` | `ModifierVisitorDemo.writeStructure(Structure structure, String filename)`             Writes a Structure to a file by using a SFWriterVisitor. |

Uses of VisitorException in org.proteinshader.structure.visitor.exceptions

| Subclasses of VisitorException in org.proteinshader.structure.visitor.exceptions | |
| --- | --- |
| `class` | `WriterVisitorException`             Used to report that an error occurred while trying to write info on a Visitable object. |

---


|  |  |  |
| --- | --- | --- |
| **Overview** \| **Package** \| **Class** \| **Use** \| **Tree** \| **Deprecated** \| **Index** \| **Help** | |  |
| PREV   NEXT | **FRAMES**    **NO FRAMES**     **All Classes** |  |


---

# *Copyright © 2007-2008*
